# Status quo of operative training in emergency surgery in Germany – results of a survey

**DOI:** 10.1007/s00423-024-03360-6

**Published:** 2024-06-20

**Authors:** N. Wachter, C. Güsgen, C. Geis, L.S. Penzkofer, K. Oldhafer, A.G. Willms, Tobias Huber

**Affiliations:** 1 Department of General, Visceral and Transplant Surgery, University Medical Center, Johannes Gutenberg-University Mainz, Langenbeckstraße 1, 55131 Mainz, Germany; 2 Department of General, Visceral and Thoracic Surgery, German Armed Forces Central Hospital, Koblenz, Germany; 3 Department of General and Visceral Surgery, German Armed Forces Hospital, Hamburg, Germany; 4 Surgical Working Group Young Surgery (CAJC) of the German Society for General and Visceral Surgery (DGAV), Berlin, Germany; 5 Surgical Working Group Military and Emergency Medicine (CAMIN) of the German Society for General and Visceral Surgery (DGAV), Berlin, Germany; 6 Department of Surgery, Clinic of HBP-Surgery, Asklepios Klinik Barmbek, Hamburg, Germany; 7 Semmelweis University of Budapest Campus, Hamburg, Germany

**Keywords:** Emergency surgery, Medical education, Surgery, Surgical sub steps, Simulation

## Abstract

**Background:**

Emergencies and emergency surgeries are a central part of everyday surgical care in Germany. However, it is unclear how emergency surgery is practically trained in clinics on a daily basis and what training concept is underlying. Therefore, the aim of this survey study was to capture the status quo of emergency surgical training of German general and visceral surgeons.

**Methods:**

The members of the German Society for General and Visceral Surgery were surveyed online (*n* = 5281). The questionnaire included demographic data and expertise in surgery and assistance in emergency surgery regarding common emergency surgical operations. In addition, further training measures in emergency surgery and their support by employers were queried.

**Results:**

Only complete questionnaires (*n* = 184, response rate 3.5%) were included in the analysis. Most participants were in training (*n* = 69; 38%), followed by senior physicians (*n* = 52; 29%), specialists (*n* = 31; 17%) and chief physicians (*n* = 30; 17%). 64% of the participants were employed at university hospitals or maximum care hospitals. Regarding further training opportunities, in-clinic shock room training was most frequently used. Outside of their own clinic, the ATLS course was most frequently mentioned. Operations for cholecystitis and appendicitis as well as emergency stoma procedures are the most common emergency procedures. There was a strong difference in the frequency of operated cases depending on the level of training. For operations to treat acute abdominal traumas (hemostasis of liver and spleen, packing) as well as outside of visceral surgery, only low competence was reported. Over 90% of survey participants consider emergency surgery to be an indispensable core competence. Neither in the old (76%) nor in the new training regulations (47%) is emergency surgery adequately represented according to the participants’ assessment. There was a significantly lower prevalence of the “sub-steps concept” in emergency surgery at 38% compared to elective surgery (44%). Important elements of imparting skills in emergency surgery are simulation and courses as well as operative sub-steps, according to the majority of survey participants.

**Conclusion:**

The results show that general and visceral surgeons in Germany are introduced to emergency surgery too little structured during further training and at specialist level. The survey participants had, as expected, hardly any experience in emergency surgery outside of visceral surgery but surprisingly also little experience in visceral surgical trauma care. There is a need to discuss the future organization of emergency surgical training. Adequate simulation structures and extracurricular courses could contribute to an improvement in this respect.

**Supplementary Information:**

The online version contains supplementary material available at 10.1007/s00423-024-03360-6.

## Introduction

Urgent or emergency operations are a central component of surgical care in both basic and regular care, as well as in maximum care hospitals or university medicine. They require emergency surgical knowledge that can extend beyond one’s own specialty. In the context of surgical training, elective procedures in one’s own specialty are usually the focus. Training assistants and trainers can be selected in advance in the context of elective surgery, and preparation individually and as a team is thus possible in terms of content and, if necessary, also practically in the sense of targeted training, e.g., of basic laparoscopic skills. With regard to emergency surgery, the model training regulations [1] for the specialist in general surgery require 20 emergency abdominal procedures (e.g., for ileus, bleeding, peritonitis, spleen rupture, hollow organ perforations), without being more precisely defined. Appendectomies also represent an urgent operation and are quantified with 25 procedures. The guideline number of 35 cholecystectomies can be performed both electively and urgently. For visceral surgery, 20 emergency procedures, 25 appendectomies, and 35 cholecystectomies are also stipulated as guideline numbers here.

In the context of urgent or emergency surgical procedures, training assistants and trainers alike face various hurdles that need to be checked before the teaching assistance can possibly be implemented. This includes (1) the content knowledge of the training assistant about the case, (2) the practical skills to manage the emergency, and (3) the circumstances of the procedure in terms of complexity, resources, and patient endangerment.

The framework conditions of the training cannot be easily transferred from elective surgery to emergency procedures. Emergency procedures often do not take place in the elective daily program, but are carried out at times of fewer resources [2], which narrows the corridor of trainees to the assistant on duty or specialist. Even with personal willingness, the Working Time Act also represents a restriction here. In addition, surgical procedures in the context of emergency care are less common, as interventional procedures, for example in the case of liver or spleen injuries, are increasingly coming to the fore [3]. Also, due to the increasing sub-specialization in various specialties, it seems to have become fundamentally more difficult to receive a broad emergency surgical education [4]. Nonetheless, there is a compelling need to acquire emergency surgical competencies both in terms of content and practical skills as part of the training. There are therefore certified courses and programs from various professional societies that are intended to compensate for this deficiency outside of everyday clinical practice. The use and later application of the knowledge from these courses has so far been reported in individual publications [5–7]. Overall, however, little is known about the extent to which these programs have found a place in everyday surgical training.

The status quo of further training in emergency surgery for general and visceral surgeons in Germany has hardly been investigated so far. For this reason, the Surgical Working Group Military and Emergency Medicine (CAMIN) and the Surgical Working Group Young Surgery (CAJC) of the German Society for General and Visceral Surgery (DGAV) have designed the present survey in collaboration on this topic.

The aim of the survey was to collect the status quo of further training in emergency surgery in Germany in order to show concepts based on this, how trainees and training assistants could optimize this accordingly.

## Material & methods

Between mid-November 2021 and the end of January 2022, a nationwide, anonymized online survey was conducted in Germany. A questionnaire was designed in advance by the study team. The link to the survey was sent on November 15, 2021, via the email addresses stored in the member database of the German Society for General and Visceral Surgery (DGAV). A reminder email was sent in January 2022 (January 17, 2022), and the survey was closed on January 31, 2022.

### Online survey

The online survey was created with the Lime-Survey © Community Edition Version 5.2.6 tool and hosted on a server at Johannes Gutenberg University Mainz. Consecutive participation in the survey was technically prevented. The questionnaire is available digitally as a supplement.

### Structure of the survey

The survey consisted of 51 questions in four blocks:

- Block A asked for personal information and one’s own level of training, as well as emergency procedures performed or assisted. This included not only typical emergency procedures in general and visceral surgery, such as appendectomies or cholecystectomies, but also a broad spectrum of emergency care, which also included procedures from other specialties such as craniotomies. The frequency of emergency procedures as a surgeon and as an assistant was recorded and categorized (0, 1–3; 4–9; 10–19; 20–39 and ≥ 40).
- Block B collected information about the hospital and the department.
- Block C aimed at offered or obligatory intra-clinical and extra-clinical further training opportunities.
- Block D recorded the individual assessment of further training in emergency surgery in general, as well as within one’s own department or hospital.

### Statistical evaluation

Prior to the study, an estimation of the population size was made to determine the necessary, meaningful sample size. In Germany, according to the Federal Medical Association in 2021, a total of 1538 general surgeons and 3457 visceral surgeons were employed in the inpatient setting [8]. In addition, there are another 3511 colleagues with the professional title “surgery”, who can also be active in the field of general and visceral surgery, totaling 8506 potential participants. Together with an only estimable number of training assistants, a population size of about 12,000 surgeons can be assumed. A sample of this size should capture the results of 163 people at a confidence interval of 90% and an error range of 5%. The data collected in the online survey were exported and imported into IBM SPSS Statistics Version 29 (International Business Machines Corporation, Armonk, NY, USA) and analyzed after cleaning. The data were first prepared descriptively. For metric variables, the presentation as absolute value with range and median was chosen. Categorical variables are shown as absolute number and as a percentage of all respondents. For further analysis of possible influencing factors such as age, gender, level of training, and position of the respondent, a Chi-square test was used. A *p* < 0.05 was considered statistically significant. Missing values were not supplemented.

## Results

### Collective

A total of 5281 members of the German Society for General and Visceral Surgery were contacted. The link contained in the invitation-email was clicked 343 times, and 184 questionnaires were completed in full. This corresponds to a response rate of 3.5%.

An overview of the demographic data of the participants is shown in Table 1. The questionnaires were mostly answered by men (*n* = 104; 57%). The median age of the participants was 38 years (range 25–69 years). Most of the participants were assistant doctors (*n* = 69; 38%), followed by senior physicians (*n* = 52; 29%), specialists (*n* = 31; 17%), and chief physicians or medical directors (*n* = 30; 17%). In the context of further training for the first desired specialist qualification, the median of the participants was in the 4th year of further training. The most frequently sought-after qualification was the specialist title for visceral surgery (*n* = 53, 29% of all persons). For specialists and senior physicians, additional further training such as Special Visceral Surgery (*n* = 29, 16% of all persons) and Special Trauma Surgery (*n* = 3, 2% of all persons) were of particular interest. The majority of chief physicians (*n* = 27, 90%) did not aspire to any further specialist qualification or additional training.

**Table 1:** Collective of survey participants (the proportional view refers to all participants who have made a statement)

Parameter		
	Absolut (n)	Proportional in [%]
sex (m : f) not specified	104 : 79 1	56,8 : 43,2 < 1
Alter [Years]	Median: 38	range: 25–69
Role in the clinic		
Chief Physician Senior Physician Specialist Trainee not specified	30 52 31 69 2	16,5 28,6 17,3 37,9
Desired Qualification (of trainees)		
Common surgery Visceral surgery Orthopedic surgery Only supplementary Education (of specialists) Common surgery Visceral surgery Orthopedic surgery Vascular surgery Thoracic surgery other Only supplementary Education No further qualification or not specified	13 46 9 1 1 7 1 1 1 1 16 87	7,1 25 4,9 0,5 0,5 3,8 0,5 0,5 0,5 0,5 8,7 47,3
Hospital level		
University Hospital Maximum Care Hospital Specialty Care Provider Primary Care Provider Not specified	64 47 35 29 9	37 27 20 17
Level of Trauma Center		
No Trauma Center Locale Trauma Center Regional Trauma Center Supra-Regional Trauma Center Not specified	7 22 44 102 9	4 13 25 58
Military Hospital		
Yes, current Yes, in the past no Not specified	20 7 149 8	11 4 85
Annual number of polytraumas in the current hospital		
0–10 11–20 21–50 51–100 > 100 Missing / Not specified	26 18 25 41 62 12	14 10 14 22 34 7

### Organization of further education and emergency surgical training

All participants (100%, *n* = 184) indicated that a rotation to an intensive care unit at their respective employer is mandatory. The rotation to the emergency room was stated as mandatory by 83% of the participants (*n* = 153). Rotations to endoscopy or other specialties were mandatory for less than a fifth (*n* = 35) or less than an eighth of the participants (*n* = 22), respectively.

**Fig. 1:**
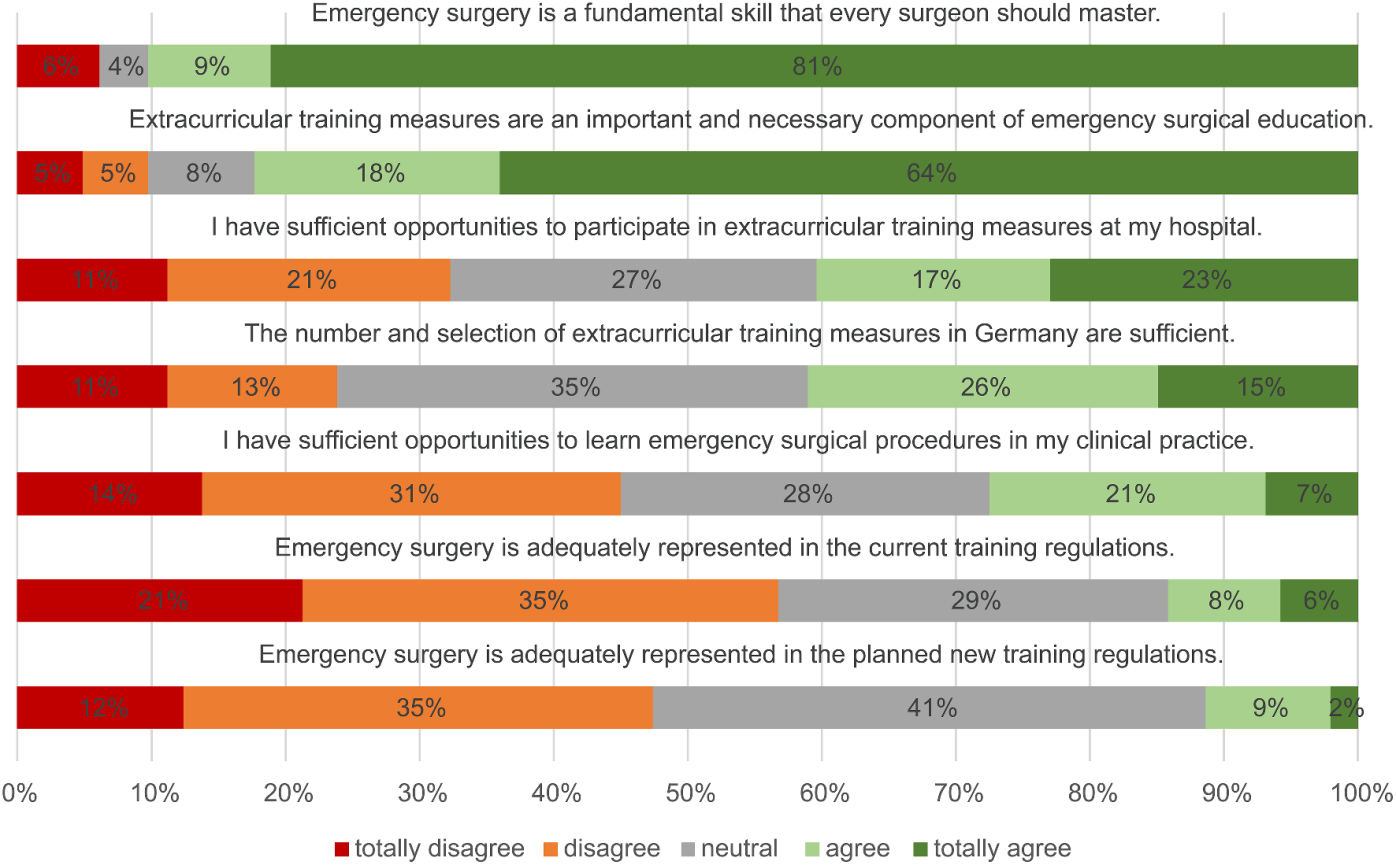
Evaluation of further education in emergency surgery, on a Likert scale from 1 = “totally disagree” to 5 = “totally agree”

An overview of the assessment of clinical and surgical training in emergency surgery is shown in Fig. 1.

**Fig. 2:**
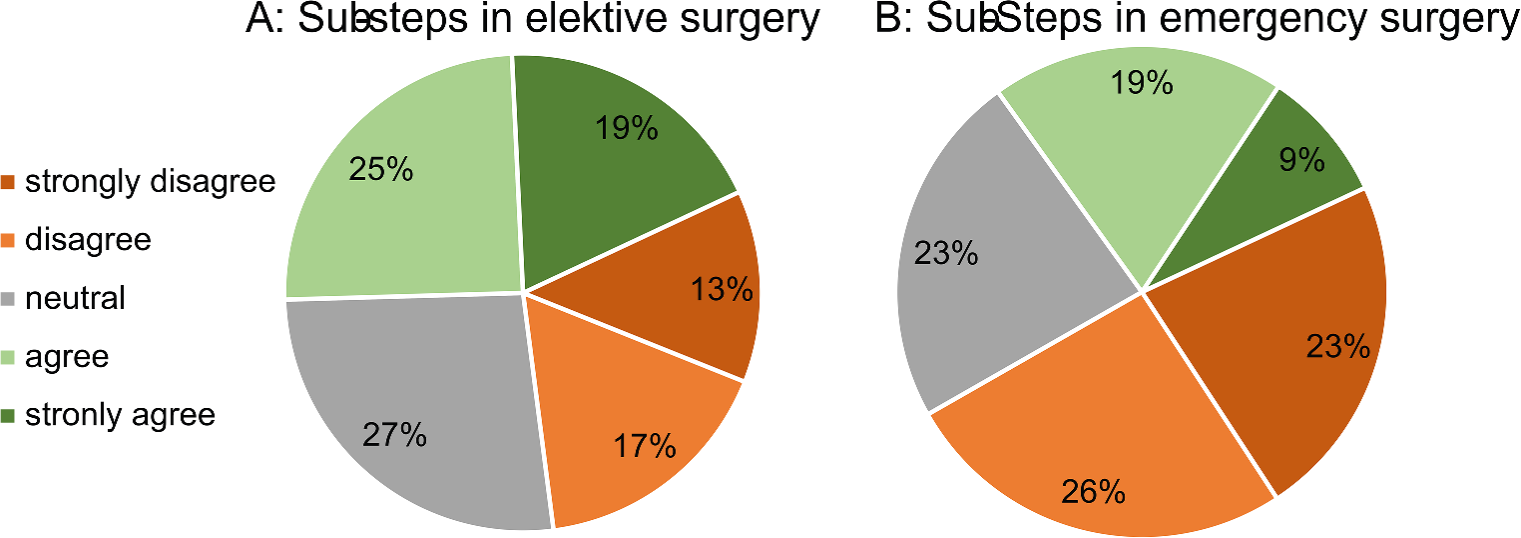
Responses to the questions: “Is the sub-step concept applied in your clinic in elective surgery (Figure A) or in emergency surgery (Figure B)?” Evaluation using a Likert scale from 1 = ?strongly disagree? to 5 = “strongly agree”

In response to the question of whether sub-steps are assisted during elective operations, 44% of participants answered “completely true” or “rather true”. For the assistance of sub-steps in emergency surgery, this percentage was significantly lower at 38% (Fig. 2). The subgroup analysis of the information on the implementation of the sub-step concept shows that training assistants reported implementation significantly less frequently than specialists. This applies to both emergency surgery (*p* = 0.003) and elective surgery (*p* = 0.006).

### Courses in the context of emergency surgical training

As part of the questionnaire, various emergency surgical training opportunities were queried, with an average of about 10% of participants not providing any information about the offer from their employer for each course.

Regarding an in-house trauma room training, half of the participants (52%, *n* = 95) responded that there is a training offer from the employer, which would be supported by most through exemption. A total of a quarter of the participants (25%, *n* = 46) stated that no in-house trauma room training is offered in their house. Another 15% (*n* = 27) could not provide any information as to whether such training exists in their operation. 36% of the participants (*n* = 66) stated that the in-house trauma room training is a training defined as obligatory by the employer.

An external course that was stated as obligatory by 35% of the participants (*n* = 65) was the Advanced Trauma Life Support (ATLS®) course from the American College of Surgeons (ACS). Almost 60% of employers (*n* = 105) supported this course, with 17% of participants (*n* = 32) also through exemption and cost absorption. About 15% of the participants (*n* = 26) could not provide any information as to whether the training is offered. Other courses from the ACS or the Royal College of Surgeons, the European Society for Trauma and Emergency Surgery or the World Society of Emergency Surgery were significantly less known and were less supported.

Only the course “Thoracoabdominal Trauma and Visceral Surgical Emergency” from CAMIN and DGAV achieved a slightly better result. A total of 33% (*n* = 60) of the participants answered that the course is recommended by the employer. 31% (*n* = 57) stated that the offer was not known.

Regarding the obligation of course participation by the employer, only the in-house trauma room training and the ATLS were each stated as mandatory by 39%.

A complete overview of the courses, offer, obligation and their participation can be found in Table 2 (Supplementary Material 1).

### Performed operations

**Fig. 3:**
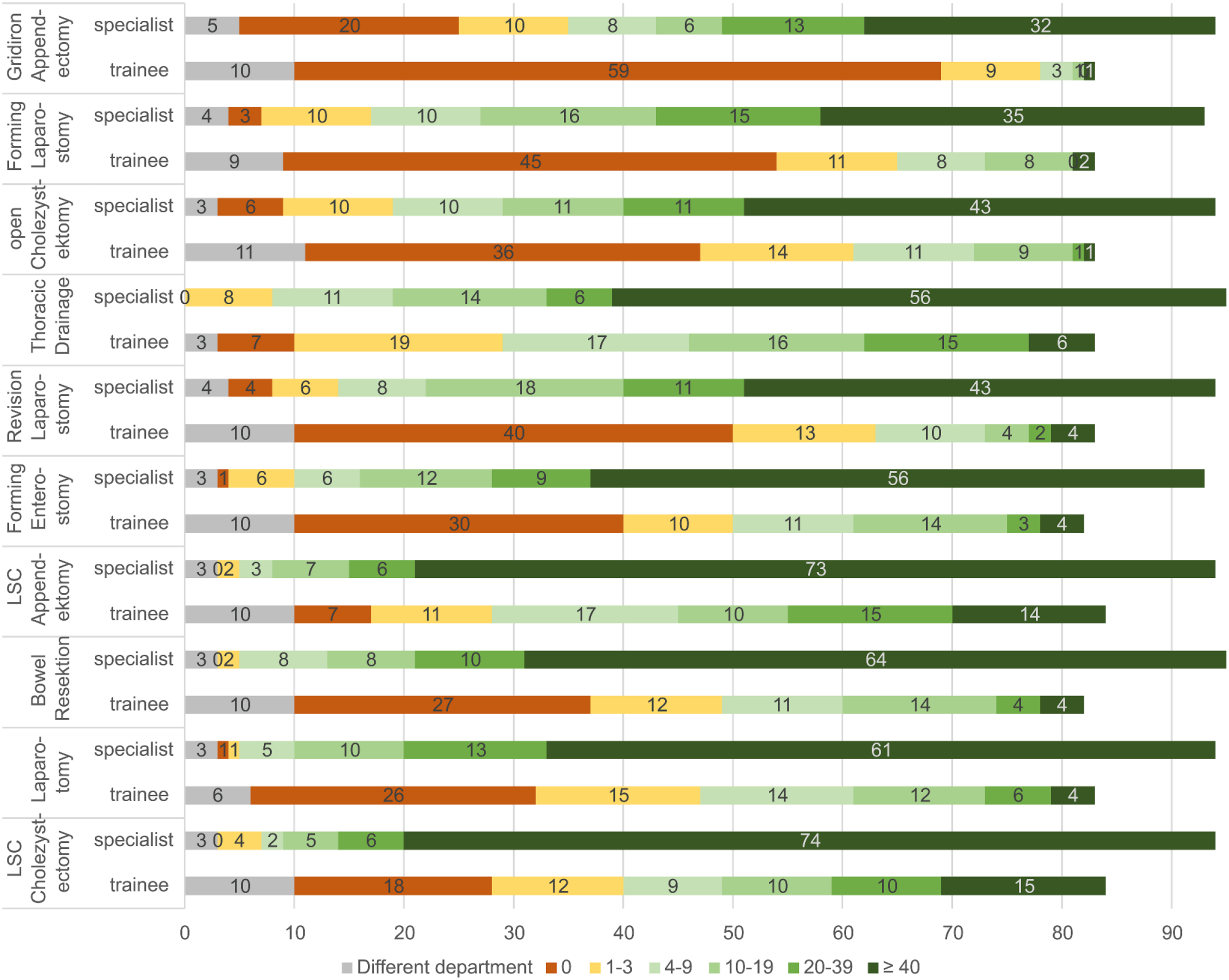
Top 10 procedures performed more than 40 times as a surgeon, broken down by training assistants vs. specialists [Numbers]. LSC: laparoscopic

The most common emergency interventions performed by themselves were typical visceral surgical indications. Fig. 3 shows the top 10 indications in their frequency according to the training level of the survey participants. As expected, it was found that the position of the participants had a significant difference on the frequency of performing an intervention (see Fig. 1). Among the associated traumatic injuries and corresponding operations, craniotomy was the rarest procedure performed, with a total of 156 people (87% of participants) stating that they had never performed a craniotomy. 145 of the participants (81%) stated that the operation was not part of their specialty and therefore was not performed.

The position of the participant had a significant influence on the frequency for each of the operations asked. Emergency laparotomies were only performed in high numbers (> 40 times) by colleagues with specialist titles, whereas almost 40% of the participating assistant doctors (*n* = 26) stated that they had never performed an emergency laparotomy.

Only the laparoscopic appendectomy (10%, *n* = 7) and the laparoscopic cholecystectomy (10%, *n* = 7) as well as the placement of a thoracic drain (5%, *n* = 3) were performed over 40 times by assistant doctors. For the specialists, a similar picture emerged: Although 42% of the specialists (*n* = 13) each stated that they had performed a laparoscopic appendectomy or a cholecystectomy more than 40 times, the frequencies for other interventions were still low. 16% of the specialists (*n* = 5) responded that they had placed an enterostoma in more than 40 emergencies. 29% of the specialists (*n* = 9) stated that they had resected the intestine in an emergency more than 40 times. Also 29% (*n* = 9) responded that they had performed an emergency laparotomy more than 40 times.

**Fig. 4:**
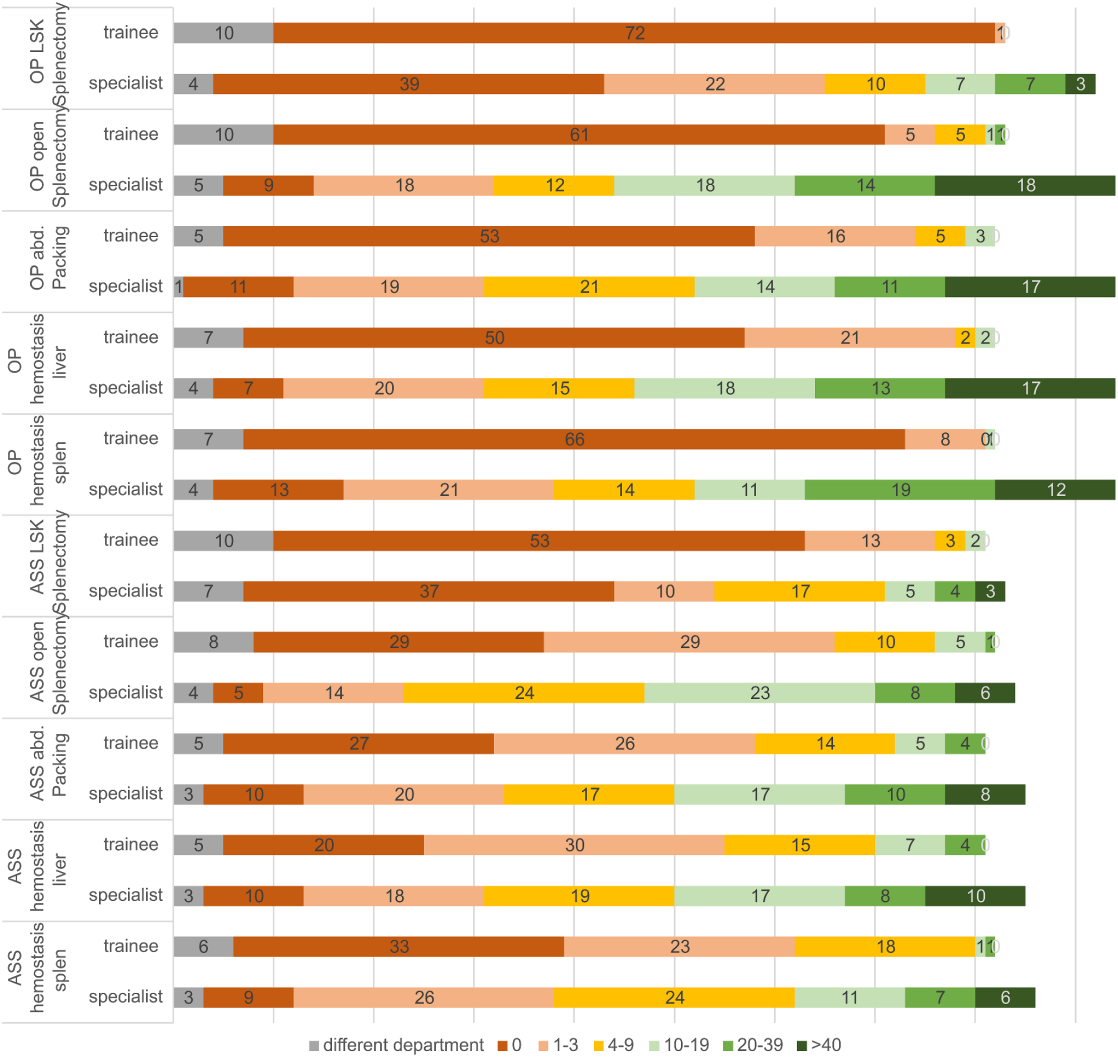
Operations following an abdominal trauma [Numbers]. Participation in an operation as Assistant (ASS) or Main Operator/Surgeon (OP)

Great experience with emergency interventions of the highest urgency such as life-threatening bleeding was reported by the fewest participants. Less than 10% of the chief physicians reported having performed these types of interventions more than 40 times. Only visceral surgical emergencies such as spleen bleeding and liver hemostasis were treated more than 40 times by more than 24% or 31% of the chief physicians. Overall, there is very little expertise in the interventions that may be required after an abdominal trauma (hemostasis on the liver or spleen, splenectomies or an abdominal packing). Fig. 4 shows these intervention frequencies stratified by specialist status and performance as an assistant or surgeon. The differences between trainee and specialist within the individual interventions were all highly significant (*p* < 0.001).

The level of care of the hospital had little influence on the frequency of performance. Although there was a tendency for participants at university hospitals and maximum care providers to report more experience with the mentioned emergency interventions, the result mostly did not reach statistical significance. Only in the subgroup analysis of bowel resections, enterostoma placements and laparoscopic cholecystectomies showed a significant influence.

**Fig. 5:**
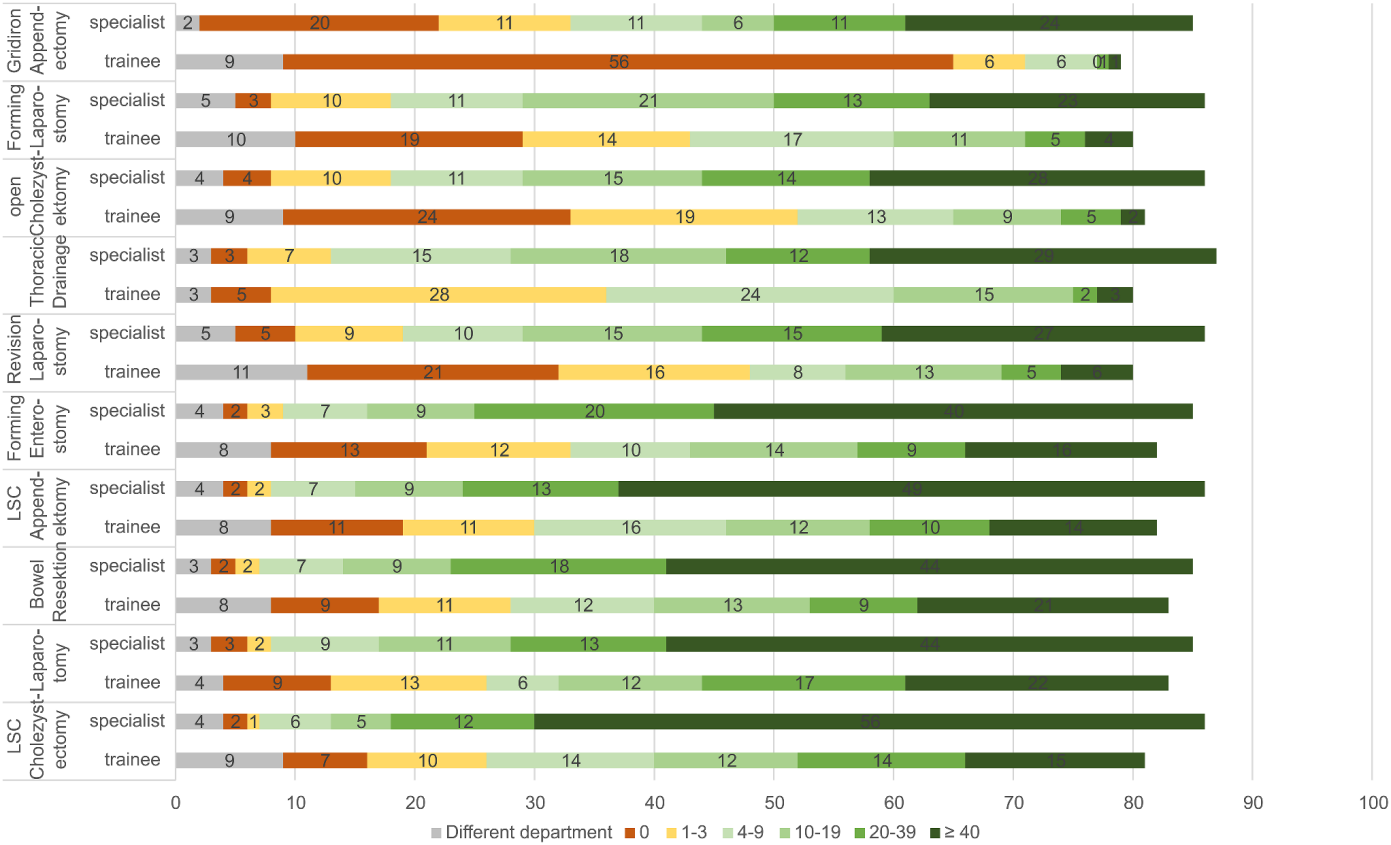
Top 10 procedures performed more than 40 times as assistant, broken down by training assistants vs. specialists [Numbers]. LSC: laparoscopic

A similar distribution was seen for assisted interventions (Fig. 5).

### Assessment of own skills and attitude towards emergency surgery

More than half of the respondents stated that they feel completely (42%, *n* = 78) or rather (25%, *n* = 45) safe with the indication for emergency interventions. The mastery of laparoscopic surgical techniques in an emergency was rated by 29% (*n* = 53) as “totally agree” and by 15% (*n* = 28) as “agree”. A total of 34% (*n* = 62) completely agreed with the statement “I master open surgery in an emergency”, another 15% (*n* = 27) rather agreed. Specialists rated themselves more secure in all questions.

Regarding the safety of the indication, there were significant differences between men and women. Men answered significantly more often with “totally agree” compared to women (54% vs. 30%, *p* = 0.009). The same was shown in the evaluation of the statements “I master laparoscopic surgery” (41% vs. 14%, *p* < 0.001), “I master open surgery” (47% vs. 18%, *p* < 0.001) and “I master the conversion to an open operation” (50% vs. 18%, *p* < 0.001). The agreement to all mentioned statements increased with the age of the respondents and the corresponding position in the clinic. Almost 90% of the participants (81% totally agree, *n* = 133; 9% agree, *n* = 15) agreed with the statement “Emergency surgery is a basic skill that every surgeon should master”. Less than 30% of the respondents (*n* = 44) stated that they have sufficient opportunities to learn emergency surgical interventions in everyday clinical practice.

## Discussion

This survey aimed to capture the status quo of emergency surgical training for general and visceral surgeons in Germany. The results show that general and visceral surgeons in Germany generally have little experience in emergency surgery outside of the visceral surgical spectrum and in interventions for abdominal trauma. In addition, only a small part of the colleagues received expertise in emergency surgery through mostly external further education. This observation contrasts with the finding that almost 90% of the participants stated that emergency surgery is a basic skill that every surgeon should master. Against the background of the geopolitical situation since February 2022, the qualifications of the Bundeswehr’s operational surgeons and the care of penetrating abdominal and thoracic traumas are also moving further into the foreground [9–11].

The results present a deficit of emergency surgical skills of German general and visceral surgeons outside of the visceral surgical area, as well as in operations to avert a potentially life-threatening situation, for example as a result of an abdominal trauma. The increasing specialization in medicine inevitably comes at the expense of a broad surgical education [3]. Interestingly, however, it was also shown that even specialist-specific emergency interventions such as acute bleeding of the liver or spleen were only treated more than 40 times by almost a third of the chief physicians. It is well known that the mortality and morbidity of a patient increases if the surgeon has little experience. As early as 2003, an analysis of more than 400,000 patient data was published in the New England Journal of Medicine, which was able to show that the mortality of the patients was inversely related to the experience of the treating surgeon [12]. More than ten years later, Mehta et al. emphasized with a retrospective analysis of more than 14,000 operations that emergency surgical patients who are operated on by surgeons who performed less than 25 operations per year had a higher mortality than patients who were operated on by surgeons who performed more than 50 operations per year [13]. The results are not surprising when you consider that skill is not only a consequence of talent, but also of practice [14]. However, if almost 40% of the participating assistant doctors stated that they had never performed an emergency laparotomy, the current further education in emergency surgery does not seem to reflect this. In order to achieve expertise, continuous further education is ultimately necessary, which can be achieved, for example, by assisting with sub-steps. In 2016, Axt et al. emphasized in a cross-sectional study that 72% of the surveyed German hospital doctors consider sub-steps to be very important [15]. It is all the more surprising at this point that less than half of the participants in our study stated that sub-steps are assisted in elective operations, as already demanded in a position paper by the CAJC in 2016 [16]. For emergency surgical interventions, the number was even lower. In addition, there is a discrepancy in the information on the sub-step concept between trainees and specialists. A 2020 published multicenter study by the CAJC showed that only a small part of about 22% of the possible sub-steps in elective index operations in visceral surgery are assisted [17]. Even in an emergency, it may be possible to assist with sub-steps in order to achieve better training. In addition, the data show that interventions that are common in elective surgery and can be performed as sub-steps of more complex interventions, such as laparotomy, revision laparotomy, thoracic drain placement, conventional cholecystectomy, enterostomy placement, are common. Thus, an implemented sub-step concept in elective surgery is also useful for further training in emergency surgery. In addition, a short “Education Team-Time-Out” can help during operation preparation (elective and emergency) to name and define the skills of the assistant [18–20]. It is not surprising that particularly younger colleagues in our study reported uncertainties regarding the performance of laparoscopic and open operations. The expertise in the area of non-specialist interventions was correspondingly even lower. Whether it is necessary for every visceral surgeon to be able to perform non-specialist emergency interventions, or whether there are specialized colleagues for certain areas, can and must be critically discussed, but can play a significant role in care away from urban centers. In this regard, there are particularly efforts in the English-speaking world to train specialized surgical colleagues who deal exclusively with emergency surgery [21]. Taking this into account, since 2016 there has been the possibility to obtain the qualification of the UEMS as part of the Fellow of the European Board of Surgery (FEBS) in Emergency Surgery. This additional qualification includes, in addition to the care of thoracic, abdominal and pelvic injuries, also the extremities, head-neck and many more. Our survey shows that this FEBS qualification would not be applicable for most survey participants, as these interventions were declared as non-specialist. However, it has also been questioned in the literature whether it might not be better for patient morbidity and mortality if, for example, in the event of a colorectal perforation, a colorectal surgeon performs the emergency operation [22]. The same applies to other areas of the body in an analogous way. The qualification of the UEMS (FEBS/EmSurg) certainly underlines the importance of emergency surgery in the overall context, but this EBSQ curriculum [23] was solely completed by a single German surgeon until 2023 [24]. Furthermore, the curriculum defines techniques, surgeries and background knowledge, however it does not define a clear pathway during surgical training for trainers and trainees, how to gradually teach emergency surgery. Other surveys also showed that emergency surgeons complain that structured concepts for the organization of emergency surgery are lacking [25, 26]. The International Federation for Emergency Medicine (IFEM) has developed a four-year curriculum for the training of emergency physicians [27] and also suggests integration into student teaching [28]. This curriculum is, however, primarily tailored to use in a central emergency department and includes all types of emergency care, including behavioral and communication training. Overall, it seems necessary in the future to discuss structured concepts for emergency surgical care in Germany [29] and to include these aspects in surgical training. Against the background of the current hospital reform and the associated restructuring of the emergency care of the individual hospital levels, this discussion becomes all the more important [30].

For the training of laparoscopic skills, there are some surveys that illuminate training enabled in-house against the background of the availability of simulators [31–33]. In Germany, these are particularly used in visceral surgery [34]. For conventional emergency surgery, the described courses exist, but a continuous training as is possible with in-house laparoscopy trainers cannot be guaranteed.

One approach to the discrepancy between the need for competence in emergency surgery and the lack of training could be - analogous to a surgical curriculum in the USA, for example - the mandatory integration of (simulation) courses into the training regulations [35, 36]. Here, of course, the question of financing by the employers, who are not yet paid for the further training of doctors in Germany, logically arises. Since 2022, the “Acute care in trauma (ACT! )” course has been held several times a year in Germany. This course represents a joint new conception of the professional societies (mainly the DGU and DGAV) on behalf of the DGCH, which bundles the previous course formats in emergency surgical training. As part of the course format, the DSTC® certificate awarded by the IATSIC, the “ASSET” certificate issued by the ACS and the actual ACT! certificate is granted. In addition, the contents of the former course for visceral surgical emergencies and thoraco-abdominal trauma are combined. Course formats like this can only represent one building block in the longitudinal acquisition of competence, but from the authors’ point of view they are hardly dispensable.

The results of our survey serve as a cornerstone for the discussion of (emergency) surgical training in Germany, but must be considered with care. Primarily members of the German Society for General and Visceral Surgery (DGAV) were surveyed, so that colleagues not being members of the DGAV could not be reached. The response rate of the survey was very low and the risk of a response or especially non-response bias is accordingly given, what represents the key limitation of the study. The sample size of 184 participants is not sufficiently representative to make a universally valid statement. Therefore, it only reflects a trend in the German society of General and Visceral Surgery. However, due to the number of participants alone, it can ultimately be statistically assumed that a valid picture of the current situation could be depicted given the overall small population size.

### Summary

The training in emergency surgery in Germany is facing major challenges due to the increasing sub-specialization and corresponding experience in surgery and assistance. The potential of the easily implementable sub-step concept in elective and emergency surgery for competence development is not being exhausted. Also, specialized simulation courses for emergency care are only limitedly supported or perceived. It is to be discussed whether such simulation courses, analogous to the European and non-European foreign countries, are to be integrated as a fixed component of surgical training.

### Electronic supplementary material

Below is the link to the electronic supplementary material.

Supplementary Material 1

## Data Availability

No datasets were generated or analysed during the current study.
