# Supplementary material for: Status quo of operative training in emergency surgery in Germany – results of a survey
Source: Langenbecks Arch Surg. 2024 Jun 20;409(1):193. doi: 10.1007/s00423-024-03360-6 (PMC11189962; doi:10.1007/s00423-024-03360-6)
Supplement: Supplementary file 1 — Supplementary Material 1 [file 423_2024_3360_MOESM1_ESM.docx]

Table 2: Supplementary Material 1: Overview of surgical emergency courses

|  | **Are the following courses offered at your clinic and how are you supported?** | | | | | | | | | | | | | | | **Obligatory** | | **Participation^2^** | |
| --- | --- | --- | --- | --- | --- | --- | --- | --- | --- | --- | --- | --- | --- | --- | --- | --- | --- | --- | --- |
|  | Not offered | | Offered, absorption of charges | | Offered, partial absorption of charges | | Offered, leave | | Offered, leave and (partial) absorption of charges e | | Offered, but no funding | | Not known | | missing |  |  |  |  |
|  | N | % | N | % | N | % | N | % | N | % | N | % | N | % | N | N | % | N | % |
| In-Hospital Trauma Bay Training (as inhouse education) | 46 | 27 | 33 | 20 | 5 | 3 | 21 | 16 | 29 | 17 | 7 | 4 | 27 | 16 | 16 | 66 | 39 | 78 | 46 |
| Advanced Trauma Life Support (ATLS®), American College of Surgeons | 37 | 22 | 46 | 27 | 9 | 5 | 11 | 7 | 32 | 19 | 7 | 4 | 26 | 15 | 16 | 65 | 39 | 68 | 40 |
| Emergency surgery course (ESC®), ESTES (European Society for Trauma and Emergency Surgery) in cooperation with the American Association for the Surgery of Trauma | 77 | 46 | 15 | 9 | 3 | 2 | 2 | 1 | 9 | 2 | 2 | 1 | 60 | 36 | 16 | 1 | <1 | 1 | <1 |
| Emergency Abdominal Surgery Course (EASC), WSES (World Society of Emergency Surgery) | 82 | 49 | 13 | 8 | 1 | <1 | 2 | 1 | 8 | 5 | 0 | 0 | 62 | 37 | 16 | - | - | - | - |
| Definitive Surgical Trauma Care (DSTC®), IATSIC (International Association for Trauma Surgery and Intensive Care) | 76 | 46 | 16 | 10 | 1 | <1 | 2 | 1 | 10 | 6 | 1 | <1 | 61 | 37 | 17 | 7 | 4 | 17 | 10 |
| Definitive Surgical Trauma Skills (DSTS), Royal College of Surgeons (UK) England | 80 | 49 | 3 | 2 | 1 | <1 | 1 | <1 | 6 | 4 | 0 | 0 | 73 | 45 | 20 | - | - | 3 | 2 |
| Thoracoabdominal Trauma and Visceral Surgical Emergency, CAMIN/DGAV | 60 | 36 | 21 | 13 | 4 | 2 | 7 | 4 | 15 | 9 | 4 | 2 | 57 | 34 | 16 | 5 | 3 | 26 | 15 |
| Advanced surgical Skills for Exposure in Trauma (ASSET), American College of Surgeons | 74 | 44 | 15 | 9 | 0 | 0 | 1 | <1 | 4 | 2 | 10 | 6 | 64 | 38 | 16 | 5 | 3 | 16 | 10 |
| Clinical Skills in Emergency Surgery, Royal College of Surgeons (UK) | 84 | 52 | 4 | 2 | 0 | 0 | 2 | 1 | 3 | 2 | 0 | 0 | 70 | 43 | 21 | - | - | 1 | <1 |
| Clinical Skills in Emergency Surgery and Trauma (SSET), Royal College of Surgeons (UK) | 84 | 50 | 5 | 3 | 0 | 0 | 1 | <1 | 4 | 2 | 0 | 0 | 73 | 44 | 17 | - | - | - | - |
| Advanced Trauma Operative Management (ATOM™), Committee on Trauma des American College of Surgeons (ACS) | 87 | 51 | 3 | 2 | 0 | 0 | 1 | <1 | 4 | 2 | 0 | 0 | 74 | 44 | 15 | - | - | - | - |

N = Number; Percentage of the valid values, rounded

^1^ Which of the following courses are mandatory for trainees and young specialists?
^2^ In which of the following courses did you participate?

Table 3: Supplementary Material 2: Surgical emergency procedures, performed as surgeon.

| Surgical procedures as (main) surgeon | Frequency of interventions (absolutely [n] and partial^6^) | | | | | | | |
| --- | --- | --- | --- | --- | --- | --- | --- | --- |
|  | DD^1^ | 0 | 1-3 | 4-9 | 10-19 | 20-39 | ≥40 | missing |
| Craniotomy | 145 (81%) | 11 (6%) | 13 (7%) | 5 (3%) | 3 (2%) | 1 (<1%) | 0 | 6 |
| Coniotomy | 81(46%) | 55 (31%) | 25 (14%) | 9 (5%) | 3 (2%) | 1 (<1%) | 2 (1%) | 8 |
| Thoracic Drainage | 3 (2%) | 7 (4%) | 29 (16%) | 28 (15%) | 30 (16%) | 21 (11%) | 64 (34%) | 2 |
| Anterolateral Thoracotomy | 51 (28%) | 54 (30%) | 21 (11%) | 16 (9%) | 15 (8%) | 11 (6) | 12 (7%) | 4 |
| Clamshell – Thoracotomy^2^ | 65 (37%) | 92 (52%) | 14 (8%) | 3 (2%) | 2 (1%) | 0 | 0 | 8 |
| Intrathoracic Hemostasis | 54 (30%) | 59 (33%) | 30 (17%) | 11 (6%) | 18 (10%) | 5 (3%) | 4 (2%) | 3 |
| External Fixator at the limbs | 99 (55%) | 16 (9%) | 10 (6%) | 22 (12%) | 11 (6%) | 8 (4%) | 14 (8%) | 4 |
| External Fixator at the pelvis | 102 (57%) | 42 (24%) | 19 (11%) | 10 (6%) | 1 (<1%) | 2 (1%) | 3 (2%) | 5 |
| Fasciotomy at the limbs | 49 (27%) | 32 (18%) | 45 (25%) | 22 (12%) | 11 (6%) | 12 (7%) | 9 (5%) | 4 |
| Abdominal Fasciotomy | 14 (8%) | 61 (34%) | 34 (19%) | 16 (9%) | 17 (10%) | 18 (10%) | 17 (10%) | 7 |
| Forming Laparostomy | 13 (7%) | 48 (34%) | 23 (13%) | 18 (10%) | 25 (14%) | 15 (8%) | 38 (21%) | 4 |
| Revision of a Laparostomy | 14 (8%) | 44 (24%) | 19 (10%) | 19 (10%) | 23 (13%) | 14 (8%) | 48 (27%) | 3 |
| Appendectomy – LSK^3^ | 13 (7%) | 7 (4%) | 13 (7%) | 20 (11%) | 17 (9%) | 24 (13%) | 88 (48%) | 2 |
| Appendectomy – GI^4^ | 15 (8%) | 80 (44%) | 21 (12%) | 12 (7%) | 7 (4%) | 13 (7%) | 33 (18%) | 3 |
| Appendectomy – other.^5^ | 16 (9%) | 58 (33%) | 25 (14%) | 24 (13%) | 19 (11%) | 14 (8%) | 22 (12%) | 6 |
| Cholezystektomie – LSK | 13 (7%) | 18 (10%) | 16 (9%) | 11 (6%) | 16 (9%) | 16 (9%) | 92 (51%) | 2 |
| Cholezystektomie – open | 14 (8%) | 42 (23%) | 25 (14%) | 22 (12%) | 21 (12%) | 12 (7%) | 45 (25%) | 3 |
| Cholezystektomie – Conversion | 13 (7%) | 56 (31%) | 33 (18%) | 22 (12%) | 23 (13%) | 11 (6%) | 23 (13%) | 3 |
| Splenectomy – LSK^3^ | 14 (8%) | 113 (63%) | 24 (13%) | 11 (6%) | 7 (4%) | 7 (4%) | 3 (2%) | 5 |
| Splenectomy – open.^6^ | 15 (8%) | 71 (39%) | 25 (14%) | 17 (9%) | 20 (11%) | 15 (8%) | 18 (10%) | 3 |
| Abdominal Packing | 6 (3%) | 64 (36%) | 38 (21%) | 27 (15%) | 17 (9%) | 11 (6%) | 17 (9%) | 4 |
| Hemostasis Liver | 11 (6%) | 57 (32%) | 43 (24%) | 19 (11%) | 20 (11%) | 13 (7%) | 17 (9%) | 4 |
| Hemostasis Spleen | 11 (6%) | 79 (44%) | 32 (18%) | 14 (8%) | 13 (7%) | 19 (11%) | 12 (7%) | 4 |
| Urgent Laparotomy | 9 (5%) | 27 (15%) | 17 (9%) | 19 (10%) | 22 (12%) | 21 (12%) | 66 (36%) | 3 |
| Bowel Resection | 13 (7%) | 27 (15%) | 14 (8%) | 19 (10%) | 23 (13%) | 17 (9%) | 68 (38%) | 3 |
| Forming Enterostomy | 13 (7%) | 31 (17%) | 16 (9%) | 17 (9%) | 29 (16%) | 13 (7%) | 60 (34%) | 5 |
| Thrombectomy / Embolectomy of the visceral vessels | 36 (20%) | 81 (46%) | 16 (9%) | 18 (10%) | 12 (7%) | 9 (5%) | 6 (3%) | 6 |
| Vessel Replacement | 60 (34%) | 69 (39%) | 13 (7%) | 9 (5%) | 14 (8%) | 5 (3%) | 8 (4%) | 6 |
| Life-threatening bleeding at the limbs | 66 (37%) | 49 (27%) | 29 (16%) | 14 (8%) | 11 (6%) | 5 (3%) | 5 (3%) | 5 |
| Life-threatening bleeding at the body-limb transitions | 50 (28%) | 64 (36%) | 33 (19%) | 10 (6%) | 14 (8%) | 4 (2%) | 3 (2%) | 6 |

^1^Different Department; ^2^both-sided Thoracotomy with horizonal Sternotomy; ^3^laparoscopic; ^4^gridiron, ^5^open, other than gridiron; ^6^valid percentage

Table 4:Supplementary Material 3: Surgical emergency procedures, performed as assistant

| Surgical procedures as assistant | Frequency of interventions (absolutely [n] and partial^6^) | | | | | | | |
| --- | --- | --- | --- | --- | --- | --- | --- | --- |
|  | Fachfremd^1^ | 0 | 1-3 | 4-9 | 10-19 | 20-39 | ≥40 | missing |
| Craniotomy | 128 (74%) | 19 (11%) | 17 (10%) | 6 (3%) | 2 (1%) | 0 | 1 (<1%) | 11 |
| Coniotomy | 92 (54%) | 45 (26%) | 23 (13%) | 8 (5%) | 3 (2%) | 0 | 0 | 13 |
| Thoracic Drainage | 6 (4%) | 9 (5%) | 35 (21%) | 39 (23%) | 33 (20%) | 14 (8%) | 32 (19%) | 16 |
| Anterolateral Thoracotomy | 41 (24%) | 32 (19%) | 29 (17%) | 27 (16%) | 22 (13%) | 11 (6%) | 9 (5%) | 13 |
| Clamshell – Thoracotomy^2^ | 64 (38%) | 91 (54%) | 12 (7%) | 2 (1%) | 0 | 0 | 1 (<1%) | 14 |
| Intrathoracic Hemostasis | 42 (24%) | 42 (24%) | 44 (26%) | 25 (15%) | 8 (5%) | 6 (3%) | 5 (3%) | 12 |
| External Fixator at the limbs | 75 (43%) | 21 (21%) | 19 (11%) | 20 (12%) | 18 (10%) | 9 (5%) | 11 (6%) | 11 |
| External Fixator at the pelvis | 80 (47%) | 47 (27%) | 19 (11%) | 14 (8%) | 6 (3%) | 4 (2%) | 2 (1%) | 12 |
| Fasciotomy at the limbs | 42 (25%) | 26 (15%) | 35 (21%) | 38 (22%) | 14 (8%) | 10 (6%) | 5 (3%) | 14 |
| Abdominal Fasciotomy | 18 (11%) | 42 (25%) | 37 (22%) | 21 (12%) | 26 (15%) | 13 (8%) | 13 (8%) | 14 |
| Forming Laparostomy | 15 (9%) | 23 (14%) | 24 (14%) | 29 (17%) | 32 (19%) | 19 (11%) | 27 (16%) | 15 |
| Revision of a Laparostomy | 16 (9%) | 26 (15%) | 26 (15%) | 19 (11%) | 28 (17%) | 20 (12%) | 34 (20%) | 15 |
| Appendectomy – LSK^3^ | 12 (7%) | 13 (8%) | 13 (8%) | 24 (14%) | 23 (13%) | 23 (13%) | 63 (37%) | 13 |
| Appendectomy – GI^4^ | 11 (7%) | 77 (46%) | 17 (10%) | 19 (11%) | 6 (4%) | 12 (7%) | 25 (15%) | 17 |
| Appendectomy – other.^5^ | 13 (8%) | 53 (31%) | 26 (15%) | 27 (15%) | 19 (11%) | 12 (7%) | 20 (12%) | 14 |
| Cholezystektomie – LSK | 13 (8%) | 9 (5%) | 11 (6%) | 21 (12%) | 19 (11%) | 26 (15%) | 71 (42%) | 14 |
| Cholezystektomie – open | 13 (8%) | 28 (16%) | 31 (18%) | 25 (15%) | 24 (14%) | 19 (11%) | 30 (18%) | 14 |
| Cholezystektomie – Conversion | 14 (8%) | 33 (19%) | 35 (21%) | 35 (21%) | 28 (16%) | 13 (8%) | 12 (7%) | 14 |
| Splenectomy – LSK^3^ | 17 (10%) | 93 (56%) | 23 (14%) | 20 (12%) | 7 (4%) | 4 (2%) | 3 (2%) | 17 |
| Splenectomy – open.^6^ | 12 (7%) | 35 (21%) | 45 (27%) | 34 (20%) | 28 (17%) | 9 (5%) | 6 (4%) | 15 |
| Abdominal Packing | 8 (5%) | 38 (22%) | 47 (28%) | 31 (18%) | 22 (13%) | 15 (9%) | 8 (5%) | 15 |
| Hemostasis Liver | 8 (5%) | 30 (18%) | 50 (30%) | 34 (20%) | 25 (15%) | 12 (7%) | 10 (6%) | 15 |
| Hemostasis Spleen | 9 (5%) | 43 (25%) | 50 (29%) | 43 (25%) | 12 (7%) | 8 (5%) | 6 (4%) | 13 |
| Urgent Laparotomy | 7 (4%) | 12 (7%) | 16 (9%) | 16 (9%) | 23 (13%) | 30 (18%) | 67 (39%) | 13 |
| Bowel Resection | 11 (6%) | 11 (6%) | 14 (8%) | 19 (11%) | 23 (13%) | 28 (15%) | 65 (38%) | 13 |
| Forming Enterostomy | 12 (8%) | 15 (9%) | 16 (9%) | 18 (11%) | 23 (14%) | 30 (18%) | 56 (33%) | 14 |
| Thrombectomy / Embolectomy of the visceral vessels | 30 (17%) | 56 (33%) | 34 (20%) | 14 (8%) | 28 (16%) | 3 (2%) | 7 (4%) | 12 |
| Vessel Replacement | 45 (26%) | 39 (23%) | 30 (17%) | 25 (14%) | 11 (6%) | 11 (6%) | 12 (7%) | 11 |
| Life-threatening bleeding at the limbs | 50 (29%) | 36 (21%) | 41 (24%) | 18 (11%) | 14 (8%) | 7 (4%) | 5 (3%) | 13 |
| Life-threatening bleeding at the body-limb transitions | 47 (27%) | 46 (27%) | 38 (22%) | 19 (11%) | 10 (6%) | 8 (5%) | 3 (2%) | 13 |

^1^Different Department; ^2^both-sided Thoracotomy with horizonal Sternotomy; ^3^laparoscopic; ^4^gridiron, ^5^open, other than gridiron; ^6^valid percentage
